# Current Clinical Practice of Precision Medicine Using Comprehensive Genomic Profiling Tests in Biliary Tract Cancer in Japan

**DOI:** 10.3390/curroncol29100573

**Published:** 2022-09-30

**Authors:** Masashi Kanai

**Affiliations:** Department of Therapeutic Oncology, Graduate School of Medicine, Kyoto University, Kyoto 606-8507, Japan; kanai@kuhp.kyoto-u.ac.jp

**Keywords:** comprehensive genomic profiling test, biliary tract cancer, precision medicine

## Abstract

With the recent advances of next generation sequencing technologies, comprehensive genomic profiling (CGP) tests, which are designed to measure more than hundreds of cancer-related genes at a time, have now been widely introduced into daily clinical practice. For the patients whose tumor samples are not fit for tissue-based CGP tests, a blood-based CGP test (liquid biopsy) is available as an alternative option. Three CGP tests, “OncoGuide NCC™Oncopanel System (124 genes)”, “FoundationOne^®^CDx (324 genes)”, and “Founda-tionOne^®^CDx Liquid (324 genes)”, are now reimbursed by public insurance in 233 hospitals designated for cancer genomic medicine in Japan. In biliary tract cancer, the prevalence of druggable variants is relatively higher compared to other cancer types and the European Society for Medical Oncology recommends routine use of CGP tests for advanced biliary tract cancer to guide treatment options. The latest National Cancer Center Network guideline lists eight druggable markers *(NTRK* fusion, MSI-H, TMB-H, *BRAF* V600E, *FGFR2* fusions/rearrangement, *IDH1* mutations, *RET* fusion, and *HER2* overexpression) and matched therapies. In Japan, matched therapies for four markers *(NTRK*, MSI-H, TMB-H, and *FGFR2*) are reimbursed by public insurance (as of September 2022). The progress of genomic profiling technology will contribute to the improvement of the dismal clinical outcomes of this disease in the future.

## 1. Introduction

Biliary tract cancer is one of the most aggressive cancers and the sixth leading cause of cancer death in Japan [1,2]. Surgery is the only curative treatment option; however, many patients are diagnosed too late for curative resection and even after curative resection, more than 50% of patients eventually develop disease recurrence [3]. For patients with unresectable or recurrent disease, systemic chemotherapy is the standard treatment option. Gemcitabine/cisplatin (GC) chemotherapy has been the global standard treatment for over a decade [4]. In addition to GC, gemcitabine/S-1 (GS) and gemcitabine/cisplatin/S-1 (GCS) are listed as the standard first-line regimens and are commonly used in Japan. In phase III trials, GS showed non-inferiority to GC [5], whereas GCS showed superiority to GC [6]. GCS was the first regimen that demonstrated survival benefits over GC in a phase III trial. Since GCS showed significantly higher response rate compared to GC (41.5% vs. 15.0%, *p* < 0.001) [6], the efficacy of GCS as a neo-adjuvant treatment for resectable disease has now been investigated in a randomized phase III trial in Japan (jRCTs031200388). A recent global phase III trial demonstrated that addition of anti-PD-L1 antibody, Durvalumab, to GC regimen significantly prolonged overall survival [7]. In spite of the recent progress of systemic chemotherapy, median survival time of patients with unresectable disease remains less than 14 months [5,6,7] and more efforts including preclinical studies have been made to improve the dismal prognosis of this disease [8].

Biliary tract cancer is a heterogeneous disease and is mainly divided into intrahepatic, perihilar, and distal subtypes according to their anatomical location. Perihilar and distal subtypes are also collectively referred to as extrahepatic disease [9]. Therefore, genomic profiling including the prevalence of druggable variants largely differs between intrahepatic and extrahepatic subtypes [10,11,12,13,14]. With the advance of next-generation sequencing (NGS) technology, comprehensive genomic profiling (CGP) tests are attracting more attention and have been introduced into daily clinical practice by several medical institutions in Japan since 2015 outside the public reimbursement system [15,16,17]. In June 2019, two CGP tests, “OncoGuide NCC™Oncopanel System (Kobe, Japan)”, “FoundationOne^®^CDx (Cambridge, MA, USA)”, were approved with reimbursement of 560,000 JPY (approximately 4300 USD). In August 2021, “FoundationOne^®^CDx Liquid” was approved with the same reimbursement system and three CGP tests are now available in 233 hospitals for cancer genomic medicine designated by the Ministry of Health, Labour and Welfare (MHLW) [18,19]. According to the registry data of the Center for Cancer Genomics and Advanced Therapeutics (C-CAT), cases of CGP tests are steadily increasing and more than 32,000 patients underwent CGP tests between June 2019 and June 2022 in Japan [20]. Biliary tract cancer is the third most common cancer type in CGP tests after colorectal and pancreatic cancer in Japan [20]. In this review article, the current clinical practice of precision medicine using CGP tests in biliary tract cancer in Japan is discussed.

## 2. Principle Flow of CGP Tests in Japan

### 2.1. Reimbursed CGP Tests

In Japan, three CGP tests, tissue–blood paired panel (OncoGuide NCC™Oncopanel System), tissue-only panel (FoundationOne^®^CDx), and liquid-based panel (FoundationOne^®^CDx Liquid), have been approved with reimbursement of 560,000 JPY (approximately 4300 USD) regardless of panel type. The characteristics of the three panels are summarized in Table 1. Reimbursement of CGP tests is limited to one time per patient.

### 2.2. Eligible Patients for CGP Tests

Eligible patients for CGP tests are restricted to those who meet the following criteria in Japan [18].

(1) Patients with locally advanced or metastatic cancer who have failed standard chemotherapy (includes patients who are expected to exhaust the standard chemotherapy regimens) or patients with rare solid tumors for which no standard chemotherapy exists.

(2) General condition is expected to remain fit for chemotherapy after the completion of CGP tests.

Due to criterion 1, patients with unresectable or recurrent biliary tract cancer cannot undergo CGP tests before initiating first-line chemotherapy in Japan. There is no scientific rationale for imposing this criterion. In fact, the latest National Cancer Center Network (NCCN) guidelines on biliary tract cancer propose molecular profiling including microsatellite instability (MSI) or tumor mutation burden (TMB) for patients with unresectable or metastatic biliary tract cancer as a routine workup before initiating first-line treatment and three molecular markers (MSI-H, *NTRK* fusion, and *RET* fusion) and matched therapies are recommended as a treatment option of first-line chemotherapy [21].

### 2.3. Requirements of Tissue Samples for Tissue-Based CGP Test

Tissue-based CGP tests usually require 10 × 5 μm slices of archival formalin-fixed paraffin-embedded (FFPE) tissue sample with ≥20% of tumor cellularity [22]. In addition, the storage period of FFPE samples is preferred to be less than 3 years to avoid DNA degradation [22,23]. Tissue sampling using cytology brushes or biopsy forceps under endoscopy is a standard method for the diagnosis of biliary tract cancer; however, these methods suffer from low sensitivity due to insufficient tissue samples or difficulties of operating biopsy forceps when the biliary stricture exists [24]. For these reasons, it is not easy to obtain sufficient tissue samples that meet the aforementioned requirements for tissue-based CGP tests in biliary tract cancer. In an earlier study, tissue-based CGP tests failed in nine (35%) out of 26 patients with biliary tract cancer due to insufficient quantity or quality of tissue samples [25]. To overcome the shortcomings of existing biopsy methods, a novel device system with improved performance of pathological diagnosis of biliary tract cancer has been developed and its clinical utility has been reported in a small cohort [24].

### 2.4. Assessment of CGP Test Results in an Expert Panel

The Ministry of Health, Labour and Welfare has designated 233 hospitals (12 core hospitals, 33 hub hospitals, and 188 affiliated hospitals) for cancer genomic medicine and CGP tests are provided under a reimbursement system in these hospitals (as of September 2022) [19]. Comprehensive Genomic Profiling test results must be reviewed by a molecular tumor board called an “Expert Panel” for reimbursement [15,18,26]. The presence of medical oncologists, pathologists, medical geneticists, genetic counselors, experts of genomic medicine, and treating physicians is required in every expert panel. Among the 233 designated hospitals for cancer genomic medicine, core hospitals (*n* = 12) and hub hospitals (*n* = 33) are eligible for holding an expert panel, whereas affiliated hospitals (*n* = 188) are not allowed to hold an expert panel independently. Therefore, affiliated hospitals have to join an expert panel held by core or hub hospitals. Comprehensive Genomic Profiling test results are returned to the treating physicians in 3 weeks on average; however, it usually takes another 2 to 4 weeks to complete the assessment of CGP test results in an expert panel. Therefore, it usually takes approximately 6 to 8 weeks for treating physicians to convey the results to patients after ordering a CGP test. For this reason, treating physicians are required to select patients who will remain fit for chemotherapy for another 6 to 8 weeks when ordering a CGP test. With the increase in CGP tests, the burden on medical staff engaged in operating an expert panel is increasing, which could lead to a delay in returning CGP test results to patients. This issue needs to be resolved as soon as possible.

### 2.5. Reimbursement System of CGP Tests in Japan

**Figure 1:**
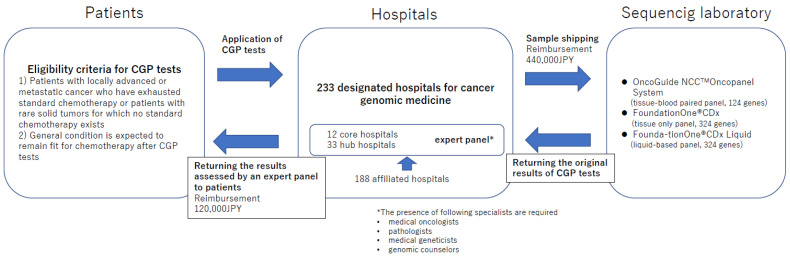
Standard flow of CGP tests in Japan.

Reimbursement of CGP tests is divided into two steps. The first reimbursement comes after shipping samples for CGP tests, whereas the second reimbursement comes after the returning of the CGP test results to patients by treating physicians. Until March 2022, the first/second reimbursement was 80,000/480,000 JPY, respectively, and hospitals could not get the second reimbursement when patients died before returning the CGP test results. According to the national insurance database, approximately 4% of patients could not get CGP test results due to deterioration of general condition or death [27]. In such cases, hospitals had to compensate the deficit of CGP test costs. To avoid the increase in financial burden of the designated hospitals for providing cancer genomic medicine CGP tests, this system has been amended since April 2022 and the current first/second reimbursement is 440,000/120,000 JPY, respectively. The principle flow of CGP tests in Japan is summarized in Figure 1.

## 3. Optimal Timing for Ordering CGP Tests in Patients with Biliary Tract Cancer

As mentioned above, ordering CGP tests before initiating first-line chemotherapy is not allowed in Japan; however, considering the dismal prognosis of biliary tract cancer, ordering CGP tests after the exhaustion of standard chemotherapy seems to be too late. National Cancer Center Network guidelines propose molecular profiling for patients with unresectable or metastatic biliary tract cancer as a routine workup before initiating first-line chemotherapy and three molecular markers (*MSI-H*, *NTRK* fusion, and *RET* fusion) and matched therapies are now recommended as a treatment option of first-line chemotherapy [21].

To investigate the clinical utility of ordering CGP tests before initiating first-line chemotherapy, two clinical trials are now underway in Japan. The FIRST-Dx trial is designed to test FoundationOne^®^CDx in 180 patients with chemotherapy-naïve advanced cancer (UMIN000042408), whereas the Upfront NCC Oncopanel Advanced medical Care trial is planned to test OncoGuide NCC™Oncopanel System in 200 patients with non-small cell lung/breast/gastric/colon/pancreatic/bile duct cancer at the initiation of systemic chemotherapy (UMIN000040743).

## 4. Liquid-Based CGP Tests (Liquid Biopsy) in Biliary Tract Cancer

### 4.1. Eligible Patients for Liquid Biopsy

As mentioned in Section 2.3, it is challenging to obtain sufficient tissue samples to meet the requirements for tissue-based CGP tests in patients with biliary tract cancer, unless primary disease is surgically resected. Repeated biopsy is not realistic in biliary tract cancer since it accompanies the risk of serious complications such as post-endoscopic retrograde cholangiopancreatography pancreatitis, infection, or bleeding [28]. For those patients whose tissue samples are too old or are disqualified for tissue-based CGP tests, liquid-based CGP tests (liquid biopsy) could be an alternative option. In Japan, FoundationOne^®^CDx Liquid has been approved for patients whose tissue samples are not fit for tissue-based CGP tests or whose tissue-based CGP tests end in failure. In addition to less invasiveness, liquid biopsy has advantages over tissue-based CGP tests in terms of capturing acquired resistant variants or tumor heterogeneity and shortening the turnaround time [29,30].

### 4.2. Performance of Liquid Biopsy in Patients with Biliary tract Cancer

In a retrospective study enrolling 124 patients with biliary tract cancer who underwent a liquid biopsy, pathogenic variants were found in 76% of patients. With regards to druggable variants, *IDH1* mutations and *BRAF* mutations were identified in 10%, and 2%, respectively [31], which was comparable with the published studies using tissue samples. In contrast, *FGFR2* fusions and *ERBB2* amplifications were identified in 2%, and 5%, respectively [31], which appeared to be lower compared with the prevalence reported in tissue samples. Several groups reported high concordance rate between tissue-based CGP and liquid biopsy in patients with solid tumors [32,33,34]; however, the concordance rate is greatly affected by platforms of liquid biopsy, target variants, tumor volumes, and the timing of blood sampling. Therefore, additional well-designed studies are warranted to clarify the concordance rate between tissue-based CGP and liquid biopsy in patients with biliary tract cancer.

### 4.3. Drawbacks of Liquid Biopsy

Treating physicians should pay attention to the following drawbacks of liquid biopsy [35].

(1) Results of MSI-H/TMB-H/copy number variations (e.g., *ERBB2* amplifications) obtained by FoundationOne^®^CDx Liquid are not approved for clinical use by MHLW in Japan.
(2) There is a risk of false negative results due to low levels of circulating tumor DNA (ctDNA), especially when total tumor volume is low.
(3) There is a risk of false positive results due to the contamination of clonal hematopoiesis with indeterminate potential (CHIP).

The number one drawback derives from MHLW regulation in Japan and is not a universal one. With regards to MSI-H, Willis et al., reported the high concordance rate of MSI-H status between tissue-based assays and liquid biopsy, if ctDNA levels were high enough. In 82 patients who were diagnosed with MSI-H using tissue assays (immunohistochemistry, polymerase chain reaction, NGS), liquid biopsy (Guardant 360) replicated MSI-H in 87% of patients [36], whereas another study reported the relatively lower prevalence of MSI-H tumors in liquid biopsy (0.7%) compared to that of tissue-based CGP tests (2.2%) [37]. Yoshino et al. also reported the relatively lower prevalence of TMB-H tumors in liquid biopsy (13%) compared to that of tissue-based CGP test (19%) [37]. In contrast, TMB results are estimated to be higher in samples with CHIP contamination [38].

To reduce the risk of false negative results, the timing of blood sampling is relevant. Since ctDNA levels are positively correlated with tumor volumes [39] and drop after response to chemotherapy [40], physicians should be cautious about the timing of blood sampling.

Clonal hematopoiesis with indeterminate potential derives from hematopoietic stem cells harboring acquired variants. The CHIP variants are commonly found in specific genes and the most common CHIP-related genes include *ATM, ASXL1, CHEK2, DNMT3A, TET2, PPM1D, JAK2,* and *TP53* [41,42]. The CHIP variants are detected in both plasma and blood cells, whereas ctDNA variants are detected only in plasma; however, since liquid biopsy measures only plasma samples, it is difficult to distinguish CHIP variants from ctDNA variants. In a retrospective study enrolling 69 patients with prostate cancer who underwent liquid biopsy, CHIP variants were contaminated in 13 patients (19%) [42]. According to the registry data of C-CAT, the top five genes reported in FoundationOne^®^CDx Liquid in patients with biliary tract cancer were *TP53, DNMT3A, ATM, CHEK2,* and *BRCA2*, whereas they were *TP53, CDKN2A, KRAS, CDKN2B,* and *ARID1A* in FoundationOne^®^CDx. Since *DNMT3A, ATM,* and *CHEK2* belong to common CHIP-related genes and are not included in the top five genes in tissue-based CGP tests, contamination of CHIP is highly suspected and variants of these genes should be interpreted with caution when liquid biopsy is applied.

### 4.4. Possible Application of Liquid Biopsy Technology for Early Detection of Biliary Tract Cancer

Application of liquid biopsy technology is not limited to CGP, but it may be helpful for early cancer detection [43]. A large prospective study, called DETECT-A (Detecting cancers Earlier Through Elective mutation-based blood Collection and Testing), has demonstrated promising results. This study screened 10,006 women who had no history of cancer by measuring plasma cell-free DNA (cfDNA) and 26 cancers were detected by this approach [44]. Several researchers are testing liquid biopsy technology for early detection of biliary tract cancer using plasma, bile juice, or urine samples [45,46,47]. Since early cancer detection could lead to curative surgery, its clinical application is warranted.

## 5. Representative Druggable Biomarkers in Biliary Tract Cancer

MOSCATO-01 is a prospective clinical trial evaluating the clinical utility of tissue-based CGP tests in a variety of cancer types [48]. In a cohort of 34 patients with biliary tract cancer, actionable variants were detected in *IDH1/2* (18%), followed by *FGFR1/2* (16%), *EGFR* or *ERBB2/3* (16%), *PTEN* (14%), *MDM2* (10%), and *PIK3CA* (10%). In total, any actionable variants were found in 23 patients (68%) and 18 patients (53%) received matched therapy, which led to a 33% of response rate [49]. The higher prevalence of actionable variants (68%) was partly attributable to the broader definition of actionable variants applied in this trial. For example, *PTEN*, *MDM2*, and *PIK3CA* are not considered to be druggable markers for patients with biliary tract cancer under the latest knowledge. As mentioned above, the latest NCCN guidelines recommend genomic profiling as a routine workup for patients with metastatic disease [21]. The ESMO Precision Medicine Working Group also recommends routine use of tissue-based CGP tests in biliary tract cancer along with three other cancer types (non-small cell lung/prostate/ovarian cancer), based on the high prevalence of druggable markers in these four cancer types [50]. Currently, eight molecular markers (*NTRK* fusion, MSI-H, TMB-H, *BRAF* V600E, *FGFR2* fusions/rearrangement, *IDH1* mutations, *HER2* overexpression, and *RET* fusions) and matched therapies are listed in the latest NCCN guidelines [21]. Among these eight molecularly targeted therapies, six are approved by the Food and Drug Administration (FDA) for biliary tract cancer, whereas four are approved by MHLW (Table 2). Although background characteristics of biliary tract cancer, including the prevalence of major druggable markers, do not largely differ between Japanese patients and those of other ethnic groups, there is a gap in drug approval between the FDA and MHLW. Matched drugs for *IDH1* mutations (ivosidenib) and *BRAF* V600E (dabrafenib plus trametinib) are awaited for approval with reimbursement by MHLW in Japan. The prevalence of druggable markers and the pivotal clinal trial results of matched therapies are summarized in Table 2.

### 5.1. MSI-H

Pembrolizumab is approved by the FDA and MHLW for patients with MSI-H in a tumor-agnostic manner based on the results of the Keynote-158 study [51]. The Keynote-158 study aimed to investigate the efficacy of pembrolizumab for patients with non-colorectal MSI-H cancer and 233 patients with 27 cancer types were enrolled. The primary endpoint of the overall response rate (ORR) was 34.3% and median overall survival (OS) was 23.5 months. In a biliary tract cancer cohort enrolling 22 patients who failed prior chemotherapy, pembrolizumab demonstrated 40.9% of ORR and 24.3 months of median OS [51]. The prevalence of MSI-H in biliary tract cancer was reported to be 1.6% (23/1017) in a nationwide large-scale investigation of microsatellite instability status [52]. MSI-H tumors are closely correlated with Lynch syndrome, which is caused by pathogenic germline variants of *MLH1, MSH2, MSH6* or *PMS2* [66]. In a retrospective study, the proportion of Lynch syndrome-associated biliary tract cancer was reported to be 0.4% (8/1899) [67].

### 5.2. NTRK Fusion

For patients with *NTRK* fusion-positive solid tumors, two TRK inhibitors, entrectinib and larotorectinib, are approved by the FDA and MHLW in a tumor-agnostic manner. In a phase I/II clinical trial enrolling 54 patients with *NTRK* fusion-positive solid tumors, entrectinib demonstrated 57% of ORR and 10 months of median duration of response [68]. Only one biliary tract cancer patient was enrolled in this study and showed partial response. In contrast, larotrectinib showed 79% of ORR in a pooled analysis of three phase I/II clinical trials (*n* = 153) [53]. Notably, complete response was observed in 24 patients (16%) and the median duration of response was 35.2 months and median OS was not reached [53]. Two patients with biliary tract cancer were enrolled and one showed partial response. The prevalence of *NTRK* fusions is estimated to be extremely rare in biliary tract cancer, and only 0.15% of patients (6/3905) harbored this variant [54]. In spite of its extremely low prevalence, NCCN guidelines now list entrectinib and larotrectinib as a treatment option for patients with *NTRK* fusion-positive biliary tract cancer at the first or subsequent-line settings [21].

### 5.3. Tumor Mutation Burden (TMB)-H

Pembrolizumab is approved by the FDA and MHLW for TMB-H (≥10 Mb) solid tumors in a tumor-agnostic manner based on the results of the KEYNOTE-158 study. In this study, pembrolizumab showed 29% of ORR (30/102) in patients with TMB-H tumors [55]; however, OS was not significantly different between TMB-H and non-TMB-H groups (11.7 months vs. 12.8 months) [55]. None of the 63 patients with biliary tract cancer enrolled in KEYNOTE-158 was diagnosed with TMB-H tumors. According to the study by Vanderwalde et al., TMB-H was found in 6 out of 177 patients with biliary tract cancer (3.4%) [56]. Among the six patients with TMB-H tumors, three patients overlapped with MSI-H tumors. In solid tumors, up to 75% of MSI-H tumors were overlapped with TMB-H tumors [56]. Therefore, the total number of patients with pure TMB-H (without MSI-H) tumors was estimated to be lower than the total number with TMB-H tumors. Several recent studies have reported that pure TMB-H tumors could not predict a good response to immune checkpoint inhibitors in several cancer types [69,70]. Further studies are warranted to clarify the true prevalence of pure TMB-H tumors and their responsiveness to immune checkpoint inhibitors in biliary tract cancer.

### 5.4. FGFR2 Fusion/Rearrangement

In a phase II study enrolling 107 patients with *FGFR2* fusion/rearrangement positive biliary tract cancer who failed prior chemotherapy (FIGHT-202), pemigatinib, an oral inhibitor of FGFR 1–3, demonstrated 35.5% of ORR, which met the predefined ORR of 33% [57]. Median progression-free survival and OS were 6.9 and 21.1 months, respectively [57]. Based on these results, pemigatinib was approved for *FGFR2* fusion/rearrangement positive biliary tract cancer by the FDA and MHLW [71]. A recent prospective observational study using fluorescent in situ hybridization (FISH) reported that the prevalence of *FGFR2* fusion/rearrangement was 5.6% (25/445) in Japanese patients with biliary tract cancer [58]. According to the study by Maruki et al., among 25 patients with *FGFR2* fusion/rearrangement-positive tumors, 21 and 4 patients had intrahepatic and perihilar disease, respectively. In contrast, *FGFR2* fusion/rearrangement were not found in 68 patients with extrahepatic disease. Thus, the prevalence of *FGFR2* fusion/rearrangement was mainly restricted to intrahepatic and perihilar disease.

### 5.5. BRAF V600E

In Rare Oncology Agnostic Research (ROAR) basket trial targeting solid tumors with *BRAF* V600E, 43 patients with *BRAF* V600E-mutated biliary tract cancer were enrolled and dabrafenib (BRAF inhibitor) plus trametinib (MEK inhibitor) showed 47% of ORR and 14 months of median OS [59]. In addition to the data from the ROAR trial, results from two other clinical trials (NCT02465060/NCT02124772) testing dabrafenib plus trametinib combination therapy on solid tumors with *BRAF* V600E were evaluated for its safety and efficacy. The overall response rate was 41% (54/131) in adult patients. Based on the data from these three clinical trials, dabrafenib plus trametinib combination therapy has recently been approved by the FDA for metastatic solid tumors harboring *BRAF* V600E in a tumor-agnostic manner [72]. In the ROAR trial, 626 patients with biliary tract cancer were prescreened for *BRAF* V600E and 57 patients (9.1%) were found to harbor this variant. *BRAF* V600E is exclusively observed in intrahepatic disease and its prevalence was reported to be 1.3% (5/377) [60] or 2.7% (11/412) [73] in other studies.

### 5.6. IDH1 Mutation

In a randomized phase III study enrolling 230 patients with *IDH1* mutated biliary tract cancer, ivosidenib, an oral inhibitor of mutated *IDH1*, significantly improved progression-free survival compared with the placebo (2.7 months vs. 1.4 months, hazard ratio 0.37, *p* < 0.0001) [61]. Although ivosidenib failed to show a significant survival benefit (10.8 months vs. 9.7 months, hazard ratio 0.69, *p* = 0.06), this drug is now approved by the FDA and is recommended for patients with *IDH1* mutation at the second or subsequent-line settings. A systematic literature review reported that the prevalence of *IDH1* activating mutation was 13.1% (552/4214) in patients with intrahepatic disease, whereas it was just 0.8% (9/1123) in patients with extrahepatic disease [62].

### 5.7. HER2 Overexpression

The efficacy of pertuzumab plus trastuzumab, both of which were anti-HER2 antibodies, was tested for patients with *HER2*-positive solid tumors who failed prior chemotherapy in Mypathway basket study. In a cohort of 39 patients with biliary tract cancer, nine patients showed partial response and ORR was 23% [63]. Median progression-free survival and OS were 4.0 months and 10.9 months, respectively. 

A recent phase II trial testing the efficacy of trastuzumab deruxtecan, an antibody–drug conjugate (ADC) drug composed of trastuzumab and topoisomerase I inhibitor, on patients with *HER2*-positive biliary tract cancer reported promising efficacy [74]. Among 22 evaluable patients with *HER2*-positive biliary tract cancer, two and six patients showed complete/partial response, respectively, and ORR was 36.4%. Although NCCN guidelines recommend trastuzumab plus pertuzumab for *HER2* positive biliary tract cancer, this combination therapy has not yet been approved by the FDA. The prevalence of *HER2* amplification was reported to be 3%/14% in intrahepatic/extrahepatic disease, respectively [64].

### 5.8. RET Fusion

In a phase I/II basket trial of *RET* inhibitor, pralsetinib, 29 patients with *RET* fusion-positive solid tumors were enrolled. ORR was 57% in 23 evaluable patients, and two out of three patients with biliary tract cancer showed partial response [65]. Median duration of response and OS were 12 months and 14 months, respectively. Authors concluded that *RET* fusion is a tumor-agnostic target for *RET* inhibitor and the latest NCCN guidelines now list pralsetinib as a treatment option for *RET* fusion-positive biliary tract cancer from the first-line setting [21]. At this time (as of September 2022), FDA approval of pralsetinib is restricted to *RET* fusion-positive non-small cell lung and thyroid cancers. The prevalence of *RET* fusion is estimated to be extremely low in biliary tract cancer and no cases were identified in 1573 patients with biliary tract cancer registered in cBioPortal [75].

### 5.9. KRAS G12C

In a phase I/II basket trial (NCT03785249) of *KRAS G12C* inhibitor adagrasib, 42 patients with *KRAS G12C* positive gastrointestinal cancers including eight biliary tract cancer cases were enrolled in a phase II cohort. Among 27 evaluable patients, ORR and disease control rate were 41%/100%, respectively [76]. At this time (as of September 2022), there are no molecularly targeted therapy recommendations for *KRAS G12C* positive biliary tract cancer in NCCN guidelines. *KRAS G12C* was identified in 0.6% (11/1573) of patients with biliary tract cancer registered in cBioPortal [75].

## 6. Germline Pathogenic Variants Identified in CGP Tests

In addition to measuring druggable markers, CGP tests can disclose the germline pathogenic variants linked with hereditary cancer risk, which are so-called “secondary findings” [77]. Since this information can potentially assist cancer screening or prevention in family members who harbor the same variants, the American College of Medical Genetics and Genomics has published a minimum gene list for reporting secondary findings in clinical sequencing [77,78]. In CCP tests, up to 10% of patients harbor pathogenic germline variants [79,80,81]. In a study enrolling 146 Japanese patients with biliary tract cancer, pathogenic germline variants were found in 11% of patients [12]. Homologous recombinant repair-related genes (e.g., *BRCA1/2, ATM*) were most common (*n* = 9), followed by Lynch syndrome-related genes (*MLH1* and *MSH2*, *n* = 3 and 1, respectively). Except for tissue–blood paired panels (e.g., OncoGuide NCC™Oncopanel System), tissue-only panels (e.g., FoundationOne^®^CDx) or liquid biopsy (e.g., FoundationOne^®^CDx Liquid) requires confirmatory germline testing for presumed germline pathogenic variants (PGPVs) to determine the true origin. However, the proportion of patients who proceed to confirmatory germline testing for PGPVs is low in Japan, probably due to the cost issue. Confirmatory germline testing is not reimbursed by public insurance and patients have to pay for the test fee out of pocket. In fact, among 2891 Japanese patients with advanced cancer who underwent FoundationOne^®^CDx, PGPVs were found in 239 patients (10%); however, less than 30% of patients with PGPVs proceeded to confirmatory germline testing, and pathogenic germline variants were confirmed in only 35 patients (1%) [82]. This figure is much lower compared to the published studies reporting an approximate 10% prevalence of pathogenic germline variants in CGP tests [79,80,81] and more patients with true pathogenic germline variants are likely to be missed in CGP tests in Japan.

## 7. Conclusions

Precision medicine using CGP tests is growing rapidly in daily clinical practice in Japan. The treatment options based on CGP are also expanding and eight molecularly matched therapies are now listed in the latest NCCN guidelines. The better understanding of underlying mechanisms of biliary tract cancer through genomic profiling will change the treatment algorism and contribute to the improvement of its dismal clinical outcome in the future.

## Figures and Tables

**Table 1:** Features of three reimbursed CGP panels in Japan.

	OncoGuide NCC™Oncopanel System	FoundationOne^®^CDx	FoundationOne^®^CDx Liquid
covered genes	124	324	324
platform	tissue and blood paired panel	tissue-only panel	blood-based panel
Results of MSI-H	available for clinical use		not available for clinical use
Results of TMB-H			
Results of CNV			
Secondary findings	no additional testing is required	confirmatory germline testing is required	
Reimbursement cost	560,000 JPY		
Approval date for reimbursement	June 2019		August 2021

**Table 2:** Druggable markers listed in NCCN guidelines.

	Matched Drug	ORR	OS	FDA Approval	MHLW Approval	Prevalence in Biliary Tract Cancer
MSI-H	Pembrolizumab	40.9% (9/20) [51]	24.3 months [51]	Yes	Yes	1.6% (23/1017) [52]
*NTRK* fusion	Entrectinib Larotrectinib	79% (121/153) [53]	not reached [53]	Yes	Yes	0.15% (6/3905) [54]
TMB-H	Pembrolizumab	29% (30/102) [55]	11.7 months [55]	Yes	Yes	3.4% (6/177) [56]
*FGFR2* fusion/ rearrangement	Pemigatinib	35.5% (38/107) [57]	21.1 months [57]	Yes	Yes	intrahepatic 7.4% (20/272) extrahepatic 2.0% (3/151) [58]
*BRAF* V600E	Dabrafenib+ Trametinib	46.5% (20/43) [59]	14 months [59]	Yes	No	intrahepatic 3% (5/159) extrahepatic 0% (0/218) [60]
*IDH1* mutaion	Ivosidenib	2% (3/124) [61]	10.8 months [61]	Yes	No	intrahepatic 13% (552/4214) extrahepatic 0.8% (9/1123) [62]
*HER2* overexpression	Pertuzumab+ Trasutuzumab	23% (9/39) [63]	10.9 months [63]	No	No	intrahepatic 3% (7/224) extrahepatic 14% (14/97) [64]
*RET* fusion	Pralsetinib	57% (13/23) [65]	14 months [65]	No	No	very low
